# First-line HIV treatment outcomes following the introduction of integrase inhibitors in UK guidelines

**DOI:** 10.1097/QAD.0000000000002603

**Published:** 2020-06-08

**Authors:** Kate El Bouzidi, Sophie Jose, Andrew N. Phillips, Anton Pozniak, Andrew Ustianowski, Mark Gompels, Alan Winston, Ab Schaap, David T. Dunn, Caroline A. Sabin

**Affiliations:** a Research Department of Infection & Population Health, Institute for Global Health; b Division of Infection & Immunity, University College London; c Chelsea and Westminster Hospital NHS Foundation Trust, London; d Pennine Acute Hospitals NHS Trust, Manchester; e North Bristol NHS Trust, Bristol; f Imperial College Healthcare NHS Trust; g Department of Infectious Disease Epidemiology, London, School of Hygiene & Tropical Medicine, London, UK.

**Keywords:** first-line antiretroviral therapy, integrase strand transfer inhibitors, integrase inhibitors, virological failure

## Abstract

**Objective:**

To investigate the characteristics and outcomes of people who initiated different antiretroviral therapy (ART) regimens during the era of integrase strand transfer inhibitors (INSTIs).

**Design:**

UK-based observational cohort study.

**Methods:**

UK Collaborative HIV Cohort study participants were included if they had started ART between 1 January 2012 and 30 June 2017. Virological failure was defined as the first of two consecutive plasma HIV RNA more than 50 copies/ml, at least 6 months after starting ART. Follow-up was censored at ART discontinuation, class switch or death. The risk of virological failure among those on INSTI, protease inhibitor or nonnucleoside reverse transcriptase inhibitor (NNRTI) regimens was compared using Kaplan–Meier and Cox regression methods.

**Results:**

Of 12 585 participants, 45.6% started a NNRTI, 29.0% a protease inhibitor and 25.4% an INSTI regimen. Over a median follow-up of 20.3 months (interquartile range 7.9–38.9), 7.5% of participants experienced virological failure. Compared with those starting an NNRTI regimen, people receiving INSTIs or protease inhibitors were more likely to experience virological failure: INSTI group adjusted hazard ratio 1.52, 95% confidence interval 1.19–1.95, *P* = 0.0009; protease inhibitor group adjusted hazard ratio 2.70, 95% confidence interval 2.27–3.21, *P* less than 0.0001, likelihood ratio test *P* less than 0.0001.

**Conclusion:**

First-line INSTI regimens were associated with a lower risk of virological failure than protease inhibitor regimens but both groups were more likely to experience virological failure than those initiating treatment with a NNRTI. There is likely to be residual channelling bias resulting from selected use of INSTIs and protease inhibitors in specific clinical contexts, including in those with a perceived risk of poor adherence.

## Introduction

Integrase strand transfer inhibitors (INSTIs) form the newest class of antiretroviral agents to be incorporated into the standard of care for treatment-naive people living with HIV in the United Kingdom. The British HIV Association (BHIVA) guidelines included raltegravir as a preferred first-line agent in 2012, followed by elvitegravir-cobicistat in 2013 and dolutegravir in 2015 [1,2]. The INSTI class has performed well when compared with other third agents in randomized controlled trials (RCTs) of first-line antiretroviral therapy (ART). The STARTMRK trial randomized ART-naive participants to receive either raltegravir or the nonnucleoside reverse transcriptase inhibitor (NNRTI) efavirenz, with a nucleos(t)ide reverse transcriptase inhibitor (NRTI) backbone of tenofovir and emtricitabine. Raltegravir was found to be noninferior to efavirenz at achieving viral suppression at 96 weeks with fewer adverse effects in the raltegravir arm (47 and 78%, *P* < 0.001) [3]. Raltegravir was then compared with the boosted protease inhibitors darunavir and atazanavir, with a tenofovir-emtricitabine backbone in the phase III open label study ACTG A5257 [4]. The incidence of virological failure was demonstrated to be equivalent for all comparisons, though the atazanavir arm had more discontinuations due to poor tolerability. The next INSTI to become available was elvitegravir, coadministered with the pharmaco-enhancing agent, cobicistat, and this was shown to be noninferior to efavirenz [5] and to atazanavir [6,7]. Dolutegravir, a next-generation INSTI, was found to be superior to efavirenz in ART-naive participants in two RCTs: SPRING-1, a phase IIb dose-ranging study in which the dolutegravir 50 mg once daily arm had 88% viral suppression at 96 weeks compared with 72% of the efavirenz arm, and SINGLE, a phase III study in which viral suppression was achieved in 88 and 81% at 48 weeks, respectively [8,9]. The difference in primary endpoint in the latter study was maintained out to week 144 (with viral suppression rates of 71 and 63% in the two groups, respectively), although interestingly the proportions with virological nonresponse at this time, as determined by the US Food and Drug Administration snapshot algorithm, demonstrated a small benefit to efavirenz (10 vs. 7%, respectively) [10]. Two further trials [11,12] demonstrated noninferiority of dolutegravir in comparison to efavirenz. Dolutegravir was also shown to be noninferior to atazanavir in the ARIA study [13] and to darunavir in the FLAMINGO study [14].

In addition to the antiretroviral efficacy demonstrated in clinical trials, the choice of regimen may depend on multiple factors that influence patient and physician preferences. These may include demands of the regimen, tolerability, toxicity, coexisting medical conditions and perceived likelihood of poor adherence. Economic considerations and accessibility are also important. Many of the older antiretroviral agents are due to come off patent in the next few years, with cheaper generic versions becoming increasingly available [15]. These factors that affect regimen selection may also be related to the effectiveness of ART. Therefore, real-world comparisons of ART classes may yield different results from those observed in clinical trial settings. The aim of this study was to investigate whether first-line ART regimens containing INSTIs are associated with a different risk of virological failure compared with other standard treatment regimens in a UK cohort of adults living with HIV. The study period of 2012–2017 encompasses the introduction of INSTIs as preferred options for first-line treatment in BHIVA guidelines and their widespread use in the United Kingdom.

## Methods

Prospectively collected data from the UK Collaborative HIV Cohort (UK CHIC) study were analysed to compare virological responses among first-line HIV treatment regimens. UK CHIC is an observational study involving 20 collaborating clinical centres, which began in 2001 with the aim of investigating clinical outcomes and treatment responses in the United Kingdom [16,17]. UK CHIC participants were included if they had initiated their first ART regimen between 1 January 2012 and 30 June 2017, allowing the potential for at least 6 months of follow-up to the end of 2017. Eligible ART regimens contained one INSTI, one boosted protease inhibitor or one NNRTI; but not more than one of these three classes. Participants were excluded if they had an undetectable viral load (HIV RNA < 50 copies/ml) at ART initiation.

### Statistical analysis

Categorical variables were compared by Chi-square test. Continuous variables with normal distributions were compared by analysis of variance (ANOVA) and those with nonnormal distributions by Kruskal–Wallis test. The main exposure of interest was the treatment group: INSTI, protease inhibitor or NNRTI. The NNRTI group was used as the reference for comparisons as this has historically been the default class of third agent recommended in the BHIVA guidelines [18–20]. The primary outcome was virological failure, which was defined as the first of two consecutive HIV RNA measurements more than 50 copies/ml, at least 6 months after ART initiation. Follow-up was censored on the date of a regimen change, date of death, 6 months after the last clinic visit or the administrative censoring date (31 December 2017), whichever was earliest. A regimen change was stopping the class that defined the treatment group (but participants could change agents within a class), or starting an agent from a different class. Participants were considered lost to follow-up on the date of the last clinic visit if this occurred more than 1 year before the administrative censoring date. Cumulative risk of virological failure was estimated by Kaplan–Meier methods, stratified by treatment group and compared with the log-rank test. Cox regression was used to estimate hazard ratios (HRs) to test the association between treatment group and virological failure, and to identify other risk factors for virological failure. An intention-to-treat analysis was performed in which a regimen change did not result in censoring follow-up. Sensitivity analysis was performed to assess the robustness of the findings to the choice of virological failure definition (e.g. single or consecutive HIV RNA measurements of >50 copies/ml, >200 copies/ml and >1000 copies/ml). All statistical analysis was undertaken with SAS software version 9.4 (SAS Institute, Cary, North Carolina, USA).

### Ethical approval

The UK CHIC study has ethical approval from the West Midlands multicentre research ethics committee (reference MREC/00/7/47) and by local ethics committees. This sub-study was approved by the UK CHIC steering committee and by the London School of Hygiene & Tropical Medicine ethics committee (reference 13628).

## Results

### Study population

**Fig. 1:**
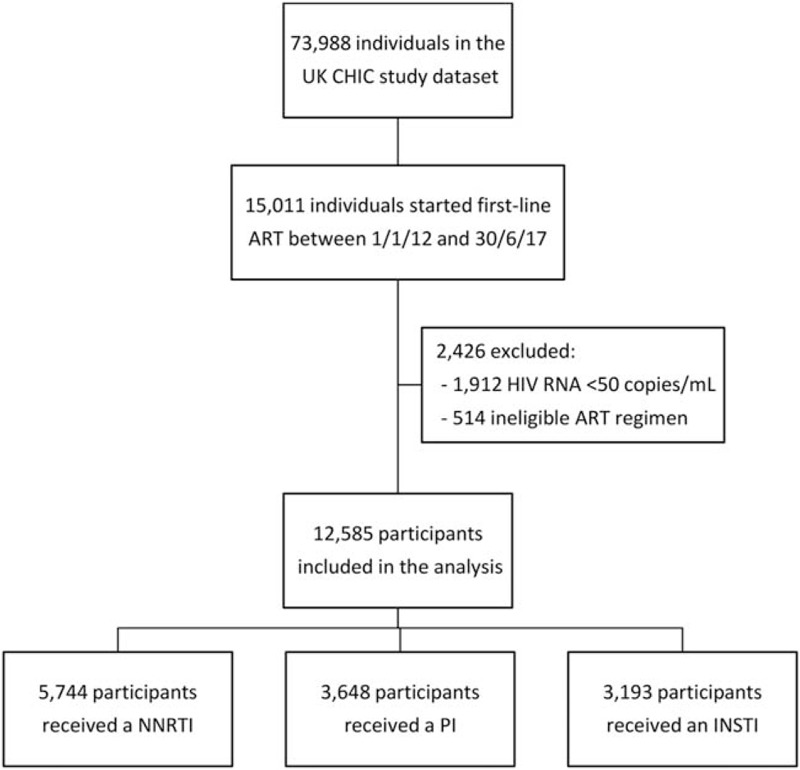
Study flow chart showing selection of eligible participants from the UK CHIC study dataset.

The UK CHIC study dataset up to the end of 2017 included 73 988 individuals, of whom 15 011 started ART between 1 January 2012 and 30 June 2017 (Fig. 1). Two thousand, four hundred and twenty-six people were excluded because they had an undetectable viral load at ART initiation or had received an ART regimen that either did not contain a NNRTI, protease inhibitor or INSTI, or contained more than one of these classes. The remaining 12 585 participants were eligible for inclusion in the study, of whom 5744 (45.6%) received a regimen containing a NNRTI, 3648 (29.0%) received a protease inhibitor and 3193 (25.4%) received an INSTI.

The baseline characteristics of the study participants are shown in Table 1. The majority were men (80%), white ethnicity (56%), with a mean age of 37 and median CD4^+^ cell count of 379 cells/μl at ART initiation. Factors independently associated with treatment group were sex, ethnicity, HIV acquisition risk group, baseline CD4^+^ cell count, viral load, year of ART initiation and NRTI backbone. In the years 2012–2013, 60.6% (3037/5010) of participants starting ART received an NNRTI; this proportion fell to 24.8% (653/2632) in 2016/2017. There was also a decrease in the proportion that received protease inhibitors, from 33.7% (1685/5010) in 2012/2013 to 24.2% (638/2632) in 2016/2017. This corresponded with the rollout of INSTIs and a rapid rise in their use as first-line agents, from 5.8% (288/5010) to 51.0% (1341/2632) in the same period. Regarding individual ART agents, the largest treatment group, NNRTI, mainly consisted of people starting efavirenz (4395/5744, 76.5%) or rilpivirine (1071, 18.7%), with a minority receiving nevirapine (247, 4.3%), etravirine (28, 0.5%) or another NNRTI (3, 0.05%). Of participants receiving a protease inhibitor, 2480 (68.0%) commenced darunavir and 1025 (28.1%) atazanavir. The older protease inhibitors, lopinavir (136, 3.7%), fosamprenavir (5, 0.1%) and saquinavir (2, 0.05%) were also prescribed in a few cases. Among those receiving an INSTI, 1886 (59.1%) received raltegravir, 935 (29.3%) dolutegravir and 372 (11.7%) elvitegravir.

**Table 1:**
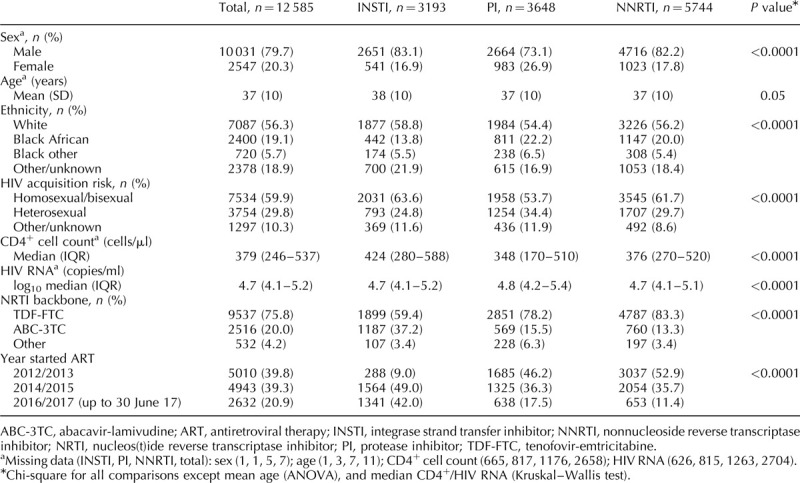
Baseline characteristics by treatment group.

### Treatment outcomes and risk factors for virological failure

**Fig. 2:**
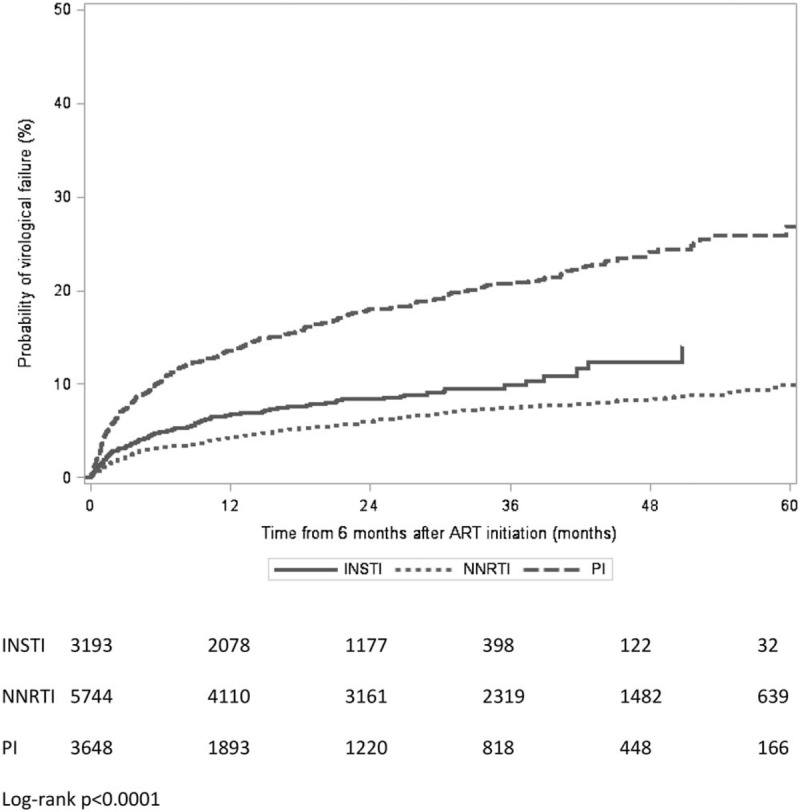
Kaplan–Meier plot showing time to virological failure, stratified by treatment group.

**Fig. 3:**
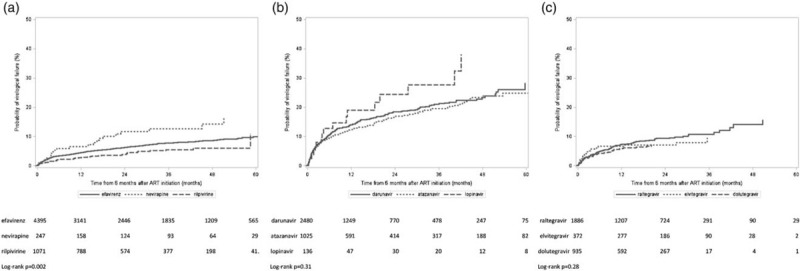
Kaplan–Meier plots showing time to virological failure stratified by agent within each treatment group: (a) nonnucleoside reverse transcriptase inhibitor, (b) protease inhibitor, (c) integrase strand transfer inhibitor.

The cohort was followed for a total of 26 067 person-years, during which time 7.5% (947/12 585) experienced virological failure. The median follow-up time on ART was 20.3 months, interquartile range 7.9–38.9 [NNRTI group: 28.3 months (10.0–48.8), protease inhibitor group: 12.7 months (6.0–33.5), INSTI group: 18.4 months (9.0–28.9), *P* < 0.0001]. Participant follow-up was censored because of a regimen change for 38.2% (36.2% of NNRTI, 56.8% of protease inhibitor and 20.4% of INSTI); at 6 months after the last clinic date for 21.1% (24.2% of NNRTI, 18.1% of protease inhibitor, 18.9% of INSTI); at death for 0.9% of the cohort (0.6% of NNRTI, 1.1% of protease inhibitor, 1.2% of INSTI) and at the end of the study period for 39.9% (39.1% of NNRTI, 24.0% of protease inhibitor, 59.5% of INSTI), *P* less than 0.0001. Overall, 13.6% (1717/12 585) of the cohort were deemed lost to follow-up, (16.0% of NNRTI, 12.5% of protease inhibitor and 10.8% of INSTI, *P* < 0.0001). Figure 2 shows the time to virological failure in the three treatment groups. In the first year of follow-up (18 months after ART initiation) the cumulative incidence curves had reached about 4% for the NNRTI group, 7% for the INSTI group and 14% for the protease inhibitor group. After 4 years of follow-up this had increased to about 8, 12 and 24%, respectively. (log-rank *P* < 0.0001). Figure 3 shows the effects of different agents within treatment groups. The NNRTI nevirapine had a higher cumulative incidence than efavirenz and rilpivirine (log-rank *P* = 0.002). The older protease inhibitor lopinavir had a higher cumulative incidence than darunavir and atazanavir, though this was a small group and the difference was not statistically significant (log-rank *P* = 0.31). The three INSTI agents had a similar virological response during the study period, although dolutegravir and elvitegravir had shorter follow-up times than raltegravir (log-rank *P* = 0.28).

Univariate Cox regression was used to examine the association between other exposure variables and virological failure. Factors with some evidence for an association with virological failure were sex [women more likely to experience virological failure than men: HR 1.45, 95% confidence interval (CI) 1.20–1.73, *P* < 0.0001]; ethnicity (black African and black other groups more likely to experience virological failure than white participants: HR 2.03, 95% CI 1.70–2.43, *P* < 0.0001 and HR 2.36, 95% CI 1.83–3.03, *P* < 0.0001, respectively); HIV acquisition risk group (heterosexual and other/unknown associated with increased virological failure compared with homosexual/bisexual: HR 1.79, 95% CI 1.53–2.10, *P* < 0.0001 and HR 1.70, 95% CI 1.30–2.22, *P* < 0.0001, respectively); baseline CD4^+^ cell count (higher CD4^+^ associated with decreased virological failure: HR 0.29 95% CI 0.24–0.37, *P* < 0.0001 for CD4^+^ > 500 cells/μl compared with CD4^+^ < 200 cells/μl); and baseline HIV RNA (high viral load associated with increased virological failure: participants with HIV RNA 100 000–1000 000 copies/ml had a HR of 2.47, 95% CI 1.65–3.71, *P* < 0.0001 and those with HIV RNA >1000 000 copies/ml had a HR of 3.54, 95% CI 2.24–5.57, *P* < 0.0001, compared with those with HIV RNA 50–1000 copies/ml). There was a trend towards lower risk of virological failure in the latter years of the study period: HR 0.72 (95% CI 0.61–0.86, *P* = 0.0002) for the 2014/2015 period and HR 0.81 (95% CI 0.64–1.03, *P* = 0.09) for the 2016/2017 period, compared with 2012/2013, likelihood ratio test *P* = 0.0007. There was no significant difference between the NRTI backbones tenofovir-emtricitabine and abacavir-lamivudine.

After adjusting for sex, ethnicity, age, HIV acquisition risk, baseline CD4^+^ cell count, HIV RNA, NRTI backbone and year of ART initiation, the INSTI and protease inhibitor groups had a higher risk of virological failure than the NNRTI group. INSTI compared with NNRTI: adjusted HR(aHR) 1.52, 95% CI 1.19–1.95, *P* = 0.0009 (unadjusted HR 1.36, 95% CI 1.10–1.68, *P* = 0.004), protease inhibitor compared with NNRTI: aHR 2.70, 95% CI 2.27–3.21, *P* less than 0.0001 (unadjusted HR 3.02, 95% CI 2.55–3.57, *P* < 0.0001), likelihood ratio test *P* less than 0.0001. The intention-to-treat analysis, in which follow-up was not censored in the case of ART class switch, showed similar results for the INSTI and protease inhibitor groups and a higher cumulative incidence in the NNRTI group (INSTI compared with NNRTI: aHR 1.18, 95% CI 0.98–1.42, *P* = 0.09; protease inhibitor compared with NNRTI: aHR 1.83, 95% CI 1.61–2.08, *P* < 0.0001). Sensitivity analyses using different definitions of virological failure showed that the number of events decreased as the threshold for failure increased, but the relative effects of treatment group were largely unchanged (Table 2). Further sensitivity analyses stratifying by calendar period and baseline CD4^+^ cell count, and also limiting follow-up time to 18 and 24 months after starting ART did not change the study findings.

**Table 2:**
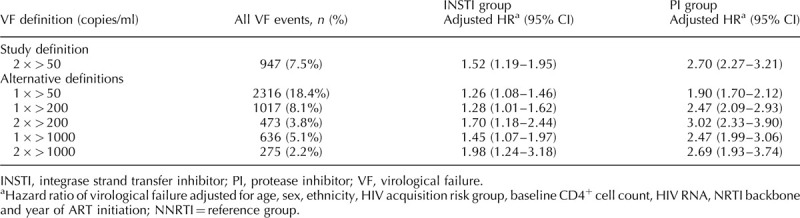
Multivariate Cox sensitivity analysis.

## Discussion

First-line ART regimens started between 2012 and 2017 in the United Kingdom were associated with a low risk of virological failure overall. People receiving protease inhibitor-containing regimens were more likely to experience virological failure than those receiving an INSTI-containing or NNRTI-containing regimen. Around a quarter of the protease inhibitor group had experienced virological failure, 4.5 years after starting ART. INSTI regimens had a lower risk of virological failure than protease inhibitors with about 12% experiencing virological failure, though this was higher than the NNRTI group at about 8%.

The treatment groups differed in many of their baseline characteristics. Although NNRTI was the most common class prescribed for men and women, women were more likely to receive a protease inhibitor regimen than men, perhaps reflecting previous concerns about the use of efavirenz during a potential childbearing period [21]. HIV acquisition risk was also strongly associated with treatment group, with heterosexual participants and those in the ‘other’ category more likely to receive protease inhibitor than homosexual or bisexual participants. This may be partly because the heterosexual group contained most of the female participants, but could also reflect that IDUs in the ‘other’ category were considered to have a higher risk of poor adherence, and so more forgiving regimens were favoured that have higher genetic barriers to resistance and also avoid efavirenz-associated central nervous system effects. The small difference in baseline CD4^+^ cell counts between treatment groups is likely to reflect the trend in recent years to starting ART at earlier stages of infection, as evidence emerged of improved clinical outcomes and reduced transmission, which coincided with the increased use of INSTIs [22–24]. These findings are similar to those of a US study that analysed the factors associated with the selection of first-line regimens from 2009 to 2012 [25]. Of 873 patients, 56% had NNRTI, 36% had protease inhibitor and 8% had raltegravir (the only INSTI available at the time). Protease inhibitors were more likely to be prescribed than NNRTIs in women [odds ratio (OR) 2.5, 95% CI 1.5–4.3]; those with baseline HIV RNA more than 100 000 copies/ml (OR 1.8, 95% CI 1.3–2.5) and active substance users (OR 1.7, 95% CI 1.2–2.5). Raltegravir was more likely to be prescribed than NNRTIs in people with a history of depression (OR 3.5, 95% CI 1.9–6.4); hepatitis C or liver disease (OR 3.3, 95% CI 1.4–7.8) and cardiovascular or cerebrovascular disease (OR 4.7, 95% CI 1.3–17.0).

There was a change in the prescribing practice during the current study period, with a decline in the proportion that received NNRTIs, and an increase in the use of protease inhibitors and INSTIs. The proportion of first-line regimens that contained INSTI increased almost 10-fold, following the availability of this class and its inclusion in BHIVA first-line treatment guidelines in 2012 [1]. Overall, 38% of participants had a regimen change, and this was more common in the protease inhibitor group and less common in the INSTI group. This is higher than observed in most RCTs, but similar to the rate of third agent change of 28 per 100 person-years (95% CI 26–31) found by a review of aggregate data from 1949 patients at eight UK centres from 2012 to 2015 [26]. It was decided to censor follow-up at a regimen change in the current study as this indicated the treatment group had changed, which may have been for economic, simplification or tolerability reasons, rather than lack of virological effectiveness.

The INSTI group had a 1.52 times greater risk of virological failure compared with NNRTI, even after adjusting for other covariates. There was a more marked difference in the protease inhibitor recipients who were 2.7 times more likely to experience virological failure. This may be because there were other factors related to poor adherence that were not measured or controlled for in this analysis. Univariate analysis suggested sex, ethnicity, HIV acquisition risk group, baseline CD4^+^ cell count, viral load and year of ART initiation were all associated with virological failure. Several other studies have identified risk factors associated with virological failure. Two analyses of UK CHIC data, spanning the periods 1996–2003 and 1998–2013, found that black ethnicity, heterosexual HIV acquisition risk group and younger age groups were associated with increased risk of virological failure [27,28]. The latter study also found earlier calendar year to be a risk factor for virological failure. The authors discussed possible reasons for the decline in virological failure over time, including nonadherent individuals leaving the at-risk population as they experienced virological failure, behaviour change to accommodate better adherence, and that viral replication may be suppressed with lower levels of adherence on established regimens [28]. A further analysis of the effect of transmitted drug resistance on first-line treatment outcomes found that those receiving protease inhibitor regimens were 2.17 times more likely to experience viraemia than those receiving NNRTI regimens (95% CI 1.88–2.51, *P* < 0.001), with no impact from transmitted resistance [29]. This study found other predictors of viraemia to be injecting drug use, black ethnicity, high baseline viral load, low CD4^+^ cell count and the use of abacavir compared with tenofovir.

One of the strengths of the current study is that it uses real-world data from a multicentre collaboration that is likely to be representative of people living with HIV in the United Kingdom, with findings that may be generalizable beyond the population that is typically recruited into randomized trials. Another advantage is that participants could switch agents within a class without their follow-up being censored, thus increasing the follow-up time to examine class effects. However, as with any observational study, our analysis may be affected by unmeasured confounding as the choice of ART class (and drugs within a class) for a given individual will be determined by many factors, including (but not limited to) information on any comorbidities present (including mental health problems) or any concomitant medications prescribed. This information may not be available in observational databases and, as a result, it may be difficult to control for these factors. Clinicians will often favour a particular ART class depending on the clinical context, even when following established treatment guidelines. For example, protease inhibitor-containing regimens and some of the newer INSTIs may be preferentially used for individuals in whom there were concerns about adherence due to the perceived higher genetic barrier to resistance of these drugs. Significantly, one of the UK CHIC contributing centres has explored the indications for raltegravir use up to the end of 2012 in treatment-naive patients, and these included the need for a rapid reduction in viral load, for example during pregnancy; concerns about drug interactions with other medication, particularly in the context of mycobacterial coinfection; and tolerability issues such as relative contraindications to efavirenz use [30]. This inability to rule out potential unmeasured confounding is the main reason why evidence from observational studies is generally rated as low quality for guideline development, although this should be balanced against the benefits, particularly related to generalizability.

Several other limitations should also be noted. In particular, UK CHIC participants were excluded if they had an undetectable viral load at ART initiation to avoid misclassification of those already receiving treatment. However, it was not possible to detect those previously treated who then present to a participating centre as ART-naive. It was surprising that 1912 people appeared to have an undetectable viral load at ART initiation, suggesting that many were already receiving ART but this information was missing from their UK CHIC record, and this group were excluded from the study. The inclusion of the newer INSTIs elvitegravir and dolutegravir, which were licensed by the European Medicines Agency in May and November of 2013 respectively, means that this study may inadvertently have included some clinical trial participants, whose responses would be likely to differ [31]. Although likely to have good internal validity to the UK population starting ART between 2012 and 2017, this study may lack generalizability to populations in other geographical settings. The findings are probably only applicable to high-income settings with a choice of ART agent. The present analysis does not include data on genotypic resistance testing; however, this will be examined in future analyses of integrase mutations associated with exposure to the INSTI class. Finally, although a small proportion of participants unfortunately died after initiating ART, we did not perform a formal competing risks analysis as the number of such deaths was small and findings were unlikely to be affected greatly by this.

In the INSTI era, first-line ART regimens containing INSTI or protease inhibitor were associated with a greater risk of virological failure than those containing NNRTI and adjusting for potential confounders did not remove this effect. Poorer virological outcomes in these groups may be related to factors associated with suboptimal adherence that have not been captured by this analysis. There is likely to be residual channelling bias resulting from selected use of INSTIs and protease inhibitors in specific clinical contexts. Furthermore, these findings illustrate the changing clinical practice in the use of first-line regimens in the United Kingdom and could be used for benchmarking of virological response in future studies.

## Acknowledgements

Authors’ contributions: K.E.B., S.J., A.N.P., A.S., D.T.D. and C.A.S. designed the study; K.E.B., S.J. and C.A.S. performed the statistical analysis; A.P., A.U., M.G. and A.W. contributed data and clinical interpretation of results; K.E.B. wrote the original draft; all authors were involved in reviewing and editing the final draft.

The UK CHIC study is funded by the UK Medical Research Council (grant numbers G0000199, G0600337, G0900274 and M004236/1). The views expressed in this article are those of the researchers and not necessarily those of the Medical Research Council. K.E.B. is supported by a Wellcome Trust clinical research fellowship (award number 170461). The data were presented at European AIDS Clinical Society (EACS) conference, 25–27 October 2017, Milan, Italy.

UK CHIC study members

*Steering Committee:* Jonathan Ainsworth, Sris Allan, Jane Anderson, David Chadwick, Duncan Churchill, Valerie Delpech, David Dunn, Richard Gilson, Mark Gompels, Phillip Hay, Teresa Hill, Margaret Johnson, Sophie Jose, Stephen Kegg, Clifford Leen, Fabiola Martin, Dushyant Mital, Mark Nelson, Chloe Orkin, Adrian Palfreeman, Andrew Phillips, Deenan Pillay, Frank Post, Jillian Pritchard, Caroline Sabin, Achim Schwenk, Anjum Tariq, Roy Trevelion, Andy Ustianowski, John Walsh.

*Central Co-ordination: University College London* (Teresa Hill, S.J., A.N.P, C.A.S., Alicia Thornton, Susie Huntington); *Medical Research Council Clinical Trials Unit at UCL, London* (D.T.D., Adam Glabay, Shaadi Shidfar).

*Participating Centres:* Barts Health NHS Trust, London (C Orkin, J Lynch, J Hand, C de Souza); Brighton and Sussex University Hospitals NHS Trust (D Churchill, N Perry, S Tilbury, E Youssef); Chelsea and Westminster Hospital NHS Foundation Trust, London (M Nelson, T Mabika, D Asboe, S Mandalia); Homerton University Hospital NHS Trust, London (J Anderson, S Munshi); King's College Hospital NHS Foundation Trust, London (F Post, A Adefisan, C Taylor, Z Gleisner, F Ibrahim, L Campbell); Middlesbrough, South Tees Hospitals NHS Foundation Trust, (D R Chadwick, K Baillie); Mortimer Market Centre, Central and North West London NHS Foundation Trust/Universtiy College London (R Gilson, N Brima, I Williams); North Middlesex University Hospital NHS Trust, London (J Ainsworth, A Schwenk, S Miller, C Wood); Royal Free NHS Foundation Trust/Universtiy Collage London (M Johnson, M Youle, F Lampe, C Smith, R Tsintas, C Chaloner, S Hutchinson, C Sabin, A Phillips, T Hill, S Jose); Imperial College Healthcare NHS Trust, London (J Walsh, N Mackie, A Winston, J Weber, F Ramzan, M Carder); The Lothian University Hospitals NHS Trust, Edinburgh (C Leen, A Wilson, S Morris); North Bristol NHS Trust (M Gompels, S Allan); Leicester, University Hospitals of Leicester NHS Trust (A Palfreeman, A Lewszuk); Woolwich, Lewisham and Greenwich NHS Trust (S Kegg, Akin Faleye, Victoria Ogunbiyi, Sue Mitchell), St. George's Healthcare NHS Trust (P Hay, C Kemble); York Teaching Hospital NHS Foundation Trust (F Martin, S Russell-Sharpe, J Gravely); Coventry, University Hospitals Coventry and Warwickshire NHS Trust (S Allan, A Harte); Wolverhampton, The Royal Wolverhampton Hospitals NHS Trust (A Tariq, H Spencer, R Jones); Chertsey, Ashford and St.Peter's Hospitals NHS Foundation Trust (J Pritchard, S Cumming, C Atkinson); Milton Keynes Hospital NHS Foundation Trust (D Mital, V Edgell, J Allen); The Pennine Acute Hospitals NHS Trust (A Ustianowski, C Murphy, I Gunder); Public Health England, London (V Delpech); i-Base (R Trevelion).

### Conflicts of interest

S.J. has received speaker's fees from Gilead Sciences. A.P. reports grants and personal fees from ViiV, Gilead, Janssen, Merck, outside the submitted work; is Principal Investigator on a Test and Treat programme in Tanzania; and is part of a research group investigating new antiretroviral regimens in South Africa. A.U. has received speaker and advisory board fees from Abbvie (in other disease areas), BMS, Gilead, Janssen, MSD and Viiv; and has received grant support from Gilead and Abbvie (in other disease areas). M.G. has received grants from BMS and Gilead to attend CROI and World AIDS 2017; all HIV companies sponsor lunches at local meetings; and has received personal fees from Advisory board to Biocryst, grants from Novartis, grants from Allergy Therapeutics, outside the submitted work. A.W. has received honoraria, been an investigator on studies sponsored by or received research grants from Gilead Sciences, GSK, BMS, Janssen-Cilag, Merck and ViiV Healthcare. D.T.D. has received honoraria, not in connection with the submitted work, from ViiV Healthcare and Gilead Sciences. C.A.S. has received honoraria from Gilead Sciences, ViiV Healthcare and Janssen-Cilag for membership of Data Safety and Monitoring Boards, Advisory Boards and for preparation of educational materials. The remaining authors have no conflicts of interest.
